# Complement deposition, IgG subtyping and endplate destruction in LRP4-ab-positive myasthenia gravis

**DOI:** 10.1186/s40478-026-02284-0

**Published:** 2026-04-21

**Authors:** Sarah Hoffmann, Katharina Brokamp, Andreas Meisel, Paolo Doksani, Lissy Helmig, Markus Schuelke, Jens-Carsten Rückert, Matthias Pumberger, Friederike Schömig, Marlene Wolfsgruber, Inga Koneczny, Marie Mayrhofer, Martin Blüthner, Tobias Ruck, Marc Pawlitzki, Werner Stenzel, Corinna Preusse

**Affiliations:** 1 Department of Neurology with Experimental Neurology, Charité – Universitätsmedizin Berlin, Freie Universität Berlin, Humboldt Universität zu Berlin, Charitéplatz 1, 10117 Berlin, Germany; 2 Department of Neuropaediatrics, Charité – Universitätsmedizin Berlin, Freie Universität Berlin and Humboldt-Universität zu Berlin, Augustenburger Platz 1, 13353 Berlin, Germany; 3 Department of Thoracic Surgery, Charité – Universitätsmedizin Berlin, Freie Universität Berlin, Humboldt Universität zu Berlin, Charitéplatz 1, 10117 Berlin, Germany; 4 Center for Musculoskeletal Surgery, Charité – Universitätsmedizin Berlin, Freie Universität Berlin and Humboldt Universität zu Berlin, Charitéplatz 1, 10117 Berlin, Germany; 5 Division of Neuropathology and Neurochemistry, Department of Neurology, Medical University of Vienna, Vienna, Austria; 6 Department of Autoimmune Diagnostics, Laboratory PD Dr. Volkmann & Colleagues SE & Co. eGbR, Medizinisches Versorgungszentrum (MVZ), Karlsruhe, Germany; 7 Department of Neurology, Ruhr University Bochum, BG University Hospital Bergmannsheil, Bochum, Germany; 8 BG University Hospital Bergmannsheil, Heimer Institute for Muscle Research, Bochum, Germany; 9 Department of Neurology, Medical Faculty, Heinrich Heine University Duesseldorf, Duesseldorf, Germany; 10 Department of Neurology, Medical Faculty, University Hospital Düsseldorf, Heinrich-Heine-University Düsseldorf, Düsseldorf, Germany; 11 Department of Neuropathology, Charité – Universitätsmedizin Berlin, Freie Universität Berlin and Humboldt-Universität zu Berlin, Charitéplatz 1, 10117 Berlin, Germany

**Keywords:** Myasthenia gravis, LRP4, Complement, C5b-9, IgG subclasses, Endplate destruction

## Abstract

Involvement of the complement system in myasthenia gravis (MG) depends on the IgG subclass of the mediating antibody. LRP4 antibodies (LRP4-ab) are typically of the IgG1 or IgG2 subclass, implying distinct capacities to activate complement. The extent and pattern of complement involvement in LRP4-ab⁺ MG remain insufficiently characterized at the human neuromuscular junction (NMJ). We examined intercostal muscle biopsies from seven patients with LRP4-ab⁺ generalized MG using immunohistochemistry, immunofluorescence including IgG-subtyping, electron microscopy (EM), and qPCR for complement-associated gene expression. AChR-ab⁺ MG patients and non-disease controls served as comparators. C5b-9 deposition at NMJs was observed in all LRP4-ab⁺ biopsies, affecting 6–96% of identified endplates. Co-localization with IgG1 was consistently detected, whereas IgG2 and IgG4 were absent. One double-seropositive patient showed focal IgG3 staining. Complement transcript levels (*C1QC*, *C3*) were higher in LRP4-ab⁺ and AChR-ab⁺ MG compared with controls. Ultrastructural analysis revealed reduced and shortened postsynaptic clefts in 3/4 evaluable LRP4-ab⁺ patients. One therapy-refractory LRP-ab + MG patient with complement deposition and endplate destruction improved after targeted complement inhibition was introduced to his therapeutic regimen, which was based on the biopsy finding. Our findings provide in situ evidence of classical complement pathway activation and associated structural alterations at the human NMJ in LRP4-ab⁺ MG. These tissue-based data contribute to a better understanding of the pathophysiology in this rare MG subgroup.

**Supplementary Information:**

The online version contains supplementary material available at 10.1186/s40478-026-02284-0.

## Introduction

Myasthenia gravis (MG) is the most prevalent neuromuscular junction (NMJ) disorder caused by autoantibodies (ab) against postsynaptic NMJ antigens. The majority of MG patients (75–80%) harbor acetylcholine receptor (AChR)-ab [1], which primarily belong to the IgG1 subtype as strong complement activators [2]. In contrast, MuSK-ab (approximately 3%) are mainly of the IgG4 subtype with only weak complement activation capacity. MuSK plays a key role in NMJ development and maintenance. MuSK-ab disrupt AChR clustering at the postsynaptic membrane [3]. This process depends on the low-density lipoprotein receptor-related protein 4 (LRP4) [4]. LRP4-ab were first implicated in MG patients without AChR- or MuSK-ab (double seronegative MG, dSN-MG) [5]. The reported prevalence of LRP4 antibodies in MG patients’ sera shows substantial variability across studies, ranging from approximately 2% up to 50%, although most studies report frequencies closer to the lower end of this spectrum in dSN-MG patients [6–8]. However, double-seropositivity of LRP4-ab with AChR- or MuSK-ab is possible [8]. While their main pathomechanism is thought to be the impairment of the agrin-LRP4-MuSK-pathway, LRP4-ab were initially mainly described as belonging to the IgG1-subclass suggesting a possible pathogenic role of the complement system [5]. However, subsequent studies reported LRP4-ab as IgG2, that activate complement less effectively [9, 10]. Targeted complement inhibition is currently restricted to AChR-ab + gMG [11–13]. Evidence of complement activation in LRP4-ab + gMG could expand their use to this patient subgroup.

LRP4-ab remain a matter of debate, as reported prevalence rates and assay sensitivities differ across studies [14, 15]. Nonetheless, several independent groups have demonstrated reproducible LRP4-antibody detection together with effector mechanisms and pathogenicity in adoptive-transfer models, supporting that LRP4 autoimmunity is biologically relevant in at least a subset of patients [9, 10, 16]. These findings underscore the importance of multimodal approaches, including structural analysis of the neuromuscular junction, to clarify the biological relevance of LRP4-ab in MG. Here, we study the role of complement on histopathological, ultrastructural and transcriptional levels including IgG-subtyping at the NMJ in intercostal muscle biopsy (ICMB) specimens of LRP4-ab+-MG patients. The intercostal muscle, rich in motor endplates, is useful for studying NMJ pathology in human neuromuscular diseases [17, 18].

## Methods

This exploratory cross-sectional study included three cohorts: seven patients with LRP4-ab⁺ gMG, disease controls consisting of ten AChR-ab⁺ gMG patients undergoing intercostal muscle biopsy (ICMB) during thymectomy, and non-disease controls (NDCs) derived from intercostal muscle samples obtained during scoliosis surgery. NDCs had no history of neuromuscular or autoimmune disease and no exposure to immunosuppressive medication. NDCs were used as morphological references and served as baseline comparators for complement transcript analyses.

LRP4-ab⁺ MG diagnosis was based on clinical presentation with fatigable muscle weakness, serological detection of LRP4 antibodies via a fixed cell–based assay (F-CBA, Labor Volkmann, Germany; to illustrate assay performance and fluorescence pattern used for interpretation, representative negative and positive control signals are shown in Supplementary Fig. 1), seronegativity for AChR and MuSK antibodies (tested via commercial ELISA, Labor Berlin) and clinical response to acetylcholinesterase inhibitors and/or immunomodulatory treatment. Archived serum samples available from 5 of the 7 patients were additionally re-tested for AChR and MuSK antibodies using live (L-CBA) and fixed cell-based assays (F-CBA) in collaboration with the Medical University of Vienna, the Methods have been described in detail elsewhere [19, 20]. Only treatment-refractory patients or those undergoing thymectomy were eligible for ICMB. At our center, we explicitly inform patients that class I evidence for thymectomy currently exists only for patients younger than 65 years with AChR-antibody–positive generalized MG and disease duration < 5 years, based on the MGTX trial [21]. In patients with LRP4-ab-^+^ or seronegative generalized MG, thymectomy may still be discussed individually when at least three of these four factors are present. This does not apply to MuSK-antibody–positive MG, in which thymectomy is not recommended. In the present cohort, all thymectomized patients fulfilled three of these factors (age < 65 years, generalized MG, disease duration < 3 years), and one patient additionally showed low-titer AChR antibodies by ELISA.

### Immunohistochemical and double immunofluorescence reactions

Muscle specimens had been cryopreserved at -80 °C prior to analysis. All stains were performed on 8 μm cryostat sections, which are stored at -20 °C.

Enzymehistochemical preparations of non-specific esterase (NSE) and acetylcholinesterase (AChE), as well as standard peroxidase-based staining of C5b-9 (DAKO, clone aE11, 1:200) was performed using standard diagnostic procedures.

For double immune stains, slides were adapted to room temperature (RT) for 20 min and subsequently fixed in acetone for 10 min. Followed by blocking with appropriate serum, followed by administration of the first primary antibody ON at 4 °C. After washing with PBS (2 × 5 min), the secondary antibody was applied. For the second antibody the procedure was repeated with incubation of the second primary antibody for one hour at RT, washing step with PBS (2 × 5 min) was followed by incubation of second secondary antibody for one hour at RT. After incubation and a final washing step (PBS 2 × 5 min), the sections were mounted with DAPI medium and stored at 4 °C. The following antibodies were used: C5b-9 (DAKO, clone aE11, 1:200) with Cy3 as secondary antibody, as well as directly coupled IgG1 Hinge-AF488 (Biozol, clone 4E3, 1:100), IgG2 Fc-AF488 (Biozol, clone HP6002, 1:100), IgG3 Hinge-AF488 (Biozol, clone HP6050, 1:100) or IgG4 Fc-AF488 (Biozol, clone HP6025, 1:100). C5b-9 (DAKO, clone aE11, 1:200) with AF488 as secondary antibody was additionally co-stained with a-Bungarotoxin-CF555. Isotype controls, secondary-ab-only or staining without primary antibody showed no immunoreactivity (see supplemental Fig. 2).

### Electron microscopy

For transmission electron microscopy (TEM), muscle biopsy specimens were fixed and embedded according to standard protocols. In brief, muscle specimens were fixed in 2.5% GA diluted in 0.1 M sodium cacodylate buffer between 24 and 72 h at 4 °C, osmicated in 1% osmium tetroxide in 0.05 M sodium cacodylate buffer, dehydrated using graded acetone series including combined en-bloc staining with 1% uranyl acetate and 0.1% phosphotungstic acid in 70% acetone, infiltrated in RenLam resin, and then polymerized for 48–72 h at 60 °C. Semithin sections (500 nm) were stained with Richardson solution (methyleneblue) for microanatomical examination, and ultrathin Sects.  (60–70 nm) were stained with uranyl acetate and lead citrate. Next, conventional ultrastructural analysis was performed using a TEM 902 (Zeiss, Oberkochen, Germany).

### Gene transcript analyses

For gene transcript analyses of complement factors via quantitative reverse transcription PCR (qRT-PCR) total RNA was extracted from whole intercostal muscle specimens via Trizol/chloroform and cDNA was synthesized using the High-Capacity cDNA Archive Kit (Applied Biosystems, Foster City, CA). For qPCR reactions, 10–20 ng of cDNA were used and for subsequent analysis, the QuantStudio 6 Flex System (Applied Biosystems, Foster City, CA) was utilized with the following running conditions: 95 °C 0:20, 95 °C 0:01, 60 °C 0:20, 45 cycles (values above 40 cycles were defined as not expressed). All targeted transcripts were investigated as technical triplicates. For each of these runs, the reference gene has been included as internal control to normalize the relative expression of the targeted transcripts. The qPCR assay identification numbers, TaqMan^®^ Gene Exp Assay from Life Technologies/ThermoFisher are listed as follows: *C1QC* Hs00757779_m1, *C3* Hs00163811_m1, *C5* Hs01004342_m1, *PGK1* Hs99999906_m1.

The ΔCT of non-diseased controls was subtracted from the ΔCT of MG patients’ muscles to determine the differences (ΔΔCT) and fold change (2^-ΔΔCT) of gene expression. Gene expression was illustrated by the fold change values compared to NDCs.

## Results

Seven LRP4-ab^+^ MG patients, ten AChR-ab^+^ MG patients and seven NDC were included. Mean age of LRP4-ab^+^ MG patients was 46.4 years (SD 23.0), 5 (71.4%) were female. Disease duration was 4.4 years (SD 3.6). Disease severity ranged from Myasthenia Gravis Foundation of America (MGFA) class IIa to V. Three patients had a history of myasthenic crisis. Three of the seven patients were double seropositive for LRP4-ab and AChR-ab (patients II, IV, V), with a single low titer positive AChR-ab result (0.6 nmol/l, 0.7 nmol/l and 0.6 nmol/l; patient II tested negative at two visits, patient IV at four visits, patient V at two visits) based on ELISA testing. None of the patients was MuSK-ab-positive by ELISA testing. None of the patients was MuSK-ab-positive by ELISA testing. 

Archived serum was available for additional L-CBA and F-CBA testing in five of the seven patients. In four cases (patients II, III, IV, V, VII), AChR and MuSK antibodies were negative in both assays. In one case (patient II), MuSK antibodies were negative in the F-CBA but showed a low positive reactivity in the L-CBA (semiquantitative score: 1 at 1:20 and 0.5 at 1:40 dilution; cut-off 0.5). The three patients with single low-titer AChR positivity by ELISA were negative for AChR antibodies in both cell-based assays. Baseline characteristics of all patients, including results from different antibody testing methods (ELISA, F-CBA, L-CBA), are presented in Table 1.

**Table 1:** Baseline characteristics of LRP4-ab + gMG patients

Patient	I	II	III	IV	V	VI	VII
Age group	EOMG	LOMG	EOMG	LOMG	EOMG	EOMG	LOMG
Sex	f	f	f	m	f	f	m
Disease duration (years)	> 3	> 3	≤ 3	> 3	≤ 3	≤ 3	> 3
RNS	–	–	–	+	–	–	–
sfEMG	n.d.	–	–	n.d.	–	–	n.d.
Response to AChE-I	+	+	+	+	+	+	–
Response to IVIg/PLEX/IA	n.d.	+	+	n.d.	+	+	+
Thymectomy ff yes, histology	– n.a.	– n.a.	+ normal	– n.a.	+ normal	+ thymic hyperplasia	– n.a.
Past myasthenic crisis	–	–	–	–	+	+	+
Current MGFA	IIa	IIIb	IIb	IIIb	IVb	IIIa	IIIa
Immunotherapy at ICMB	no	interval IVIg	GCS	GCS, AZA	interval IVIg	GCS, AZA	GCS, RTX, EFG
Antibody Status							
LRP4-ab (F-CBA)	+	+	+	+	+	+	+
AChR-ab (ELISA)	–	low +	–	low +	low +	–	–
AChR-ab (L-CBA)	s.n.a.	–	–	–	–	s.n.a.	–
AChR-ab (F-CBA)	s.n.a.	–	–	–	–	s.n.a.	–
MuSK-ab (ELISA)	–	–	–	–	–	–	–
MuSK-ab (L-CBA)	s.n.a.	low +	–	–	–	s.n.a.	–
MuSK-ab (F-CBA)	s.n.a.	–	–	–	–	s.n.a.	–

ab=antibody; AChE-I=acetylcholine esterase inhibitor; L-CBA=live cell-based assay; F-CBA=fixed cell-based assay; RNS=repetitive nerve stimulation; sfEMG=single-fiber electromyography; ICMB=intercostal muscle biopsy; IVIg=Intravenous immunoglobulins; PLEX=plasma exchange; IA=immunoadsorption; MGFA=Myasthenia Gravis Foundation of America classification; GCS=glucocorticosteroids; AZA=azathioprine; MMF=mycophenolate mofetil; MTX=methotrexate; EFG=efgartigimod; n.d.=not given/ performed; n.a.= not applicable; s.n.a.=serum not available; +=positive result; *–*=negative result. To ensure data anonymization in accordance with journal requirements, patient age was categorized into early-onset myasthenia gravis (EOMG; onset < 50 years) and late-onset myasthenia gravis (LOMG; onset ≥ 50 years)

**Fig. 1:**
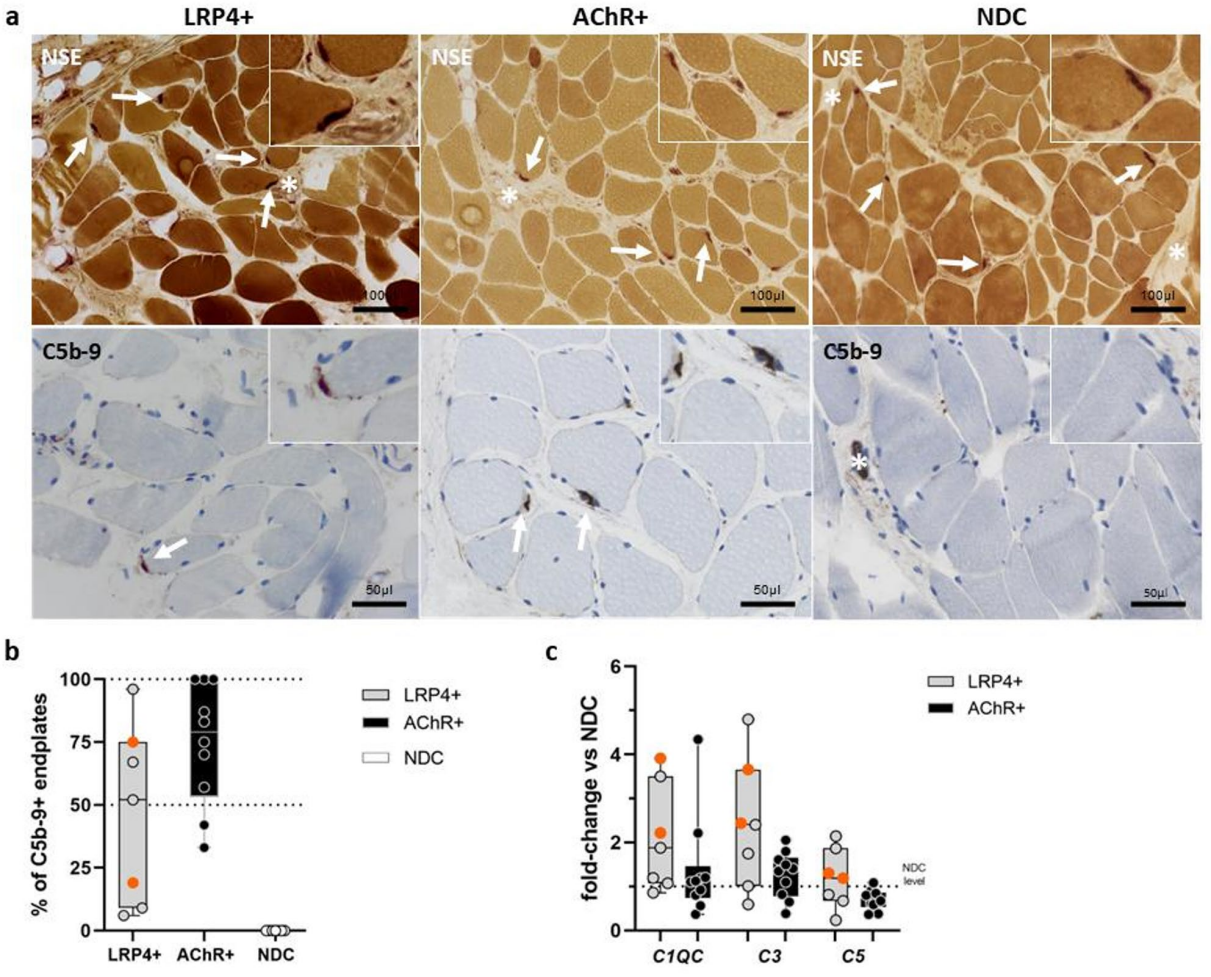
Complement deposition at NMJ and gene expression of complement factors. **a** Enzyme histochemical preparation by non-specific esterase shows motor endpoint regions with terminal nerve bundles (asterisk) and neuromuscular junctions (NMJ, white arrows) in LRP4-ab + and AChR-ab+-MG patients as well as non-diseased controls (NDC). Only in myasthenia gravis (MG) patients, NMJ are stained positive for C5b-9. Note, physiological positivity of small arterioles (with a lumen) in NDC muscle tissues, while endplates are not stained. **b** Counting of C5b-9 positive endplates in the whole slide demonstrated a range of 6–96% and a mean of 46% C5b-9^+^ endplates in LRP4-ab + MG patients (double positive patients marked in orange), while in AChR+ patients, range was 33–100% with a mean of 75%. **c** Gene transcript analyses of complement factors via qPCR demonstrated increased expression of *C1QC* and *C3* in MG patients without reaching statistical significance. Statistics with Ordinary one-way ANOVA with Tukey´s multiple comparison test or Mann-Whitney U test, *p* < 0.05. LRP4/AChR-double positive patients marked in orange

Endplates were identified by non-specific esterase (NSE), acetylcholine esterase (AChE) and CD56. C5b-9 staining (membrane attack complex, MAC) at NMJs was observed in all LRP4-ab⁺ MG biopsies, with proportions ranging from 6% to 96% (mean 46%) of identified NMJs (Fig. 1a, b). C5b-9 staining was also observed in all AChR-ab⁺ MG patients (range 33–100%, mean 75%). NDCs did not show C5b-9 staining at NMJs (Fig. 1a). Expression levels of the complement factors *C1QC* and *C3* were higher in LRP4-ab⁺ and *C3* levels were also higher in AChR-ab⁺ MG compared to NDC, though this finding was not statistically significant (Fig. 1c).

**Fig. 2:**
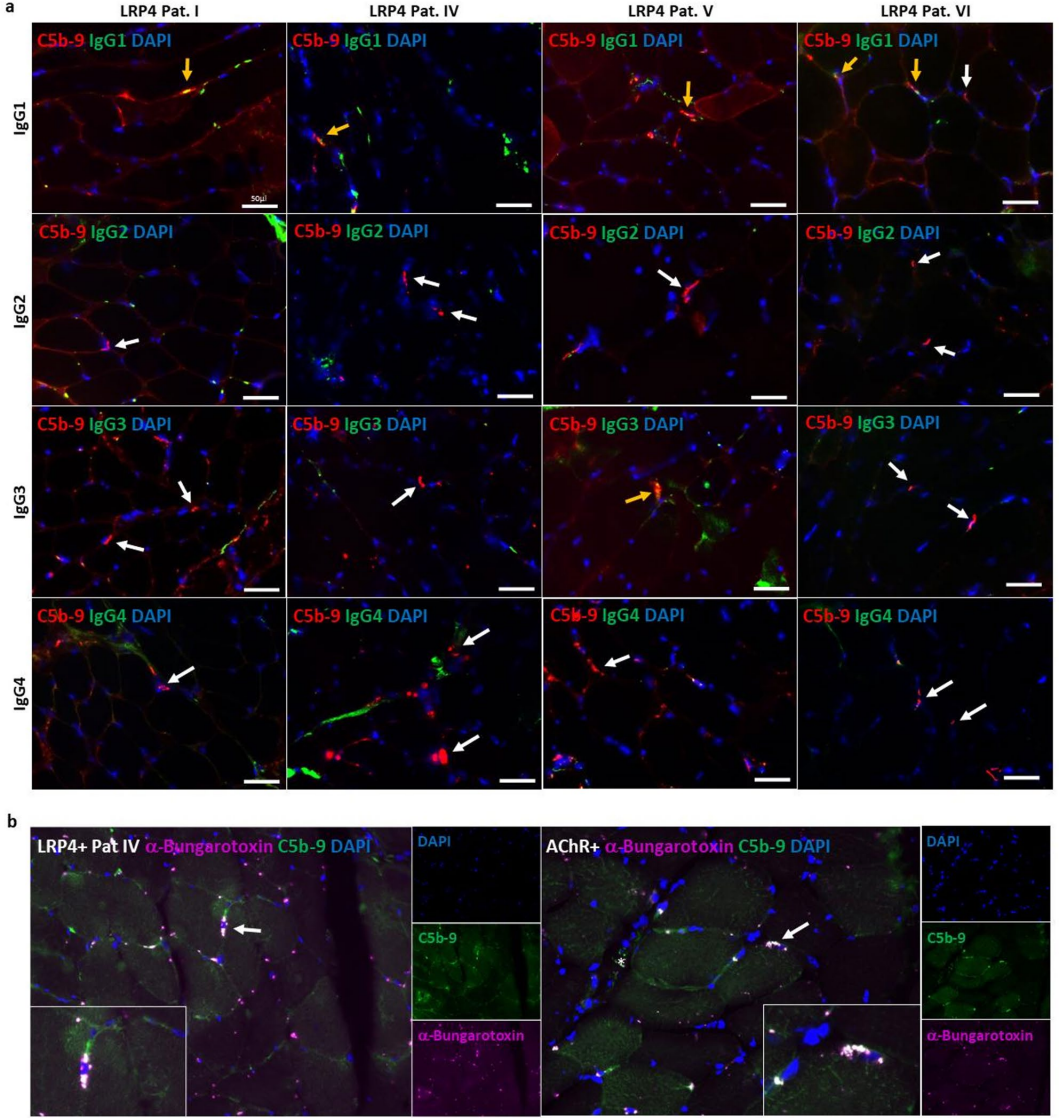
IgG subtyping in LRP4-ab-positive MG. **a** LRP4-ab^+^-patients showed co-localization of MAC (C5b-9) with IgG1, and one of the double-seropositive LRP4-ab^+^/AChR-ab^+^ MG patients (patient V) showed additional positivity for IgG3 on single endplates. There was no IgG2 or IgG4 staining at the NMJ of LRP4-ab^+^ MG patients. C5b-9 staining = red, IgG subclasses = green, nuclear staining by DAPI = blue, double-positivity of C5b-9 and IgGs = yellow arrow, no double-positivity = white arrow, scale bar = 50 μm; **b** exemplary picture of one LRP4 + and one AChR+ patient, showing co-localisation of MAC (C5b-9) with neuromuscular junctions stained by α-Bungarotoxin; C5b-9 staining = green, α-Bungarotoxin = purple, nuclear staining by DAPI = blue, double-positivity of C5b-9 and α-Bungarotoxin = white color and white arrow, magnification 200x

**Fig. 3:**
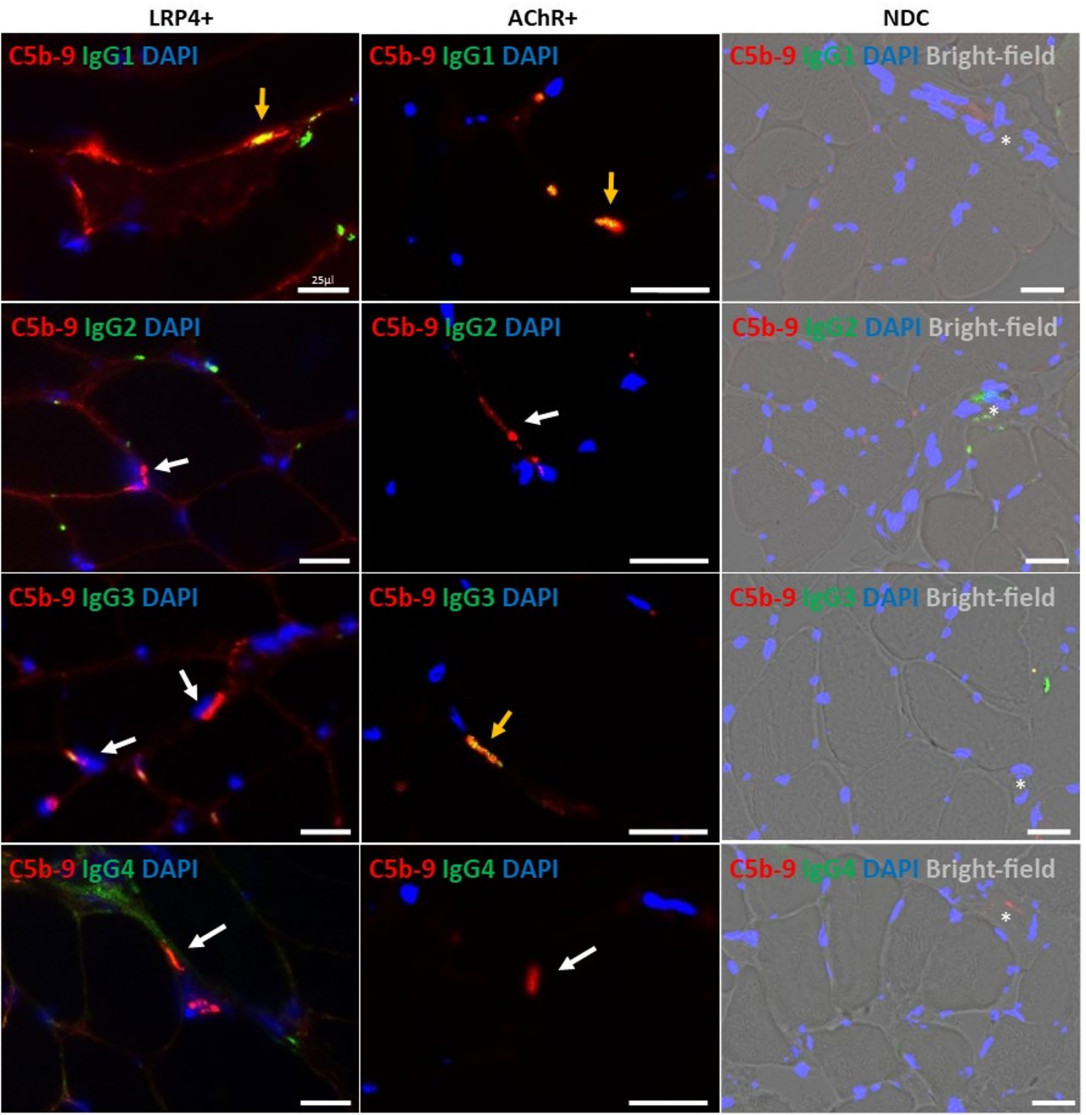
C5b-9 deposition and IgG subclass patterns in LRP4-ab⁺ and AChR-ab⁺ MG and in non-diseased controls. In LRP4-ab^+^ MG, a consistent co-localization of MAC/C5b-9 with IgG1 could be detected, while there was no IgG2 or IgG4 staining at the NMJ. One of the double-seropositive LRP4-ab^+^/AChR-ab^+^ MG patients (patient V) showed additional positivity for IgG3 on single endplates (depicted in Fig. +/AChR2). In AChR-ab + MG a consistent co-localization of MAC with IgG1 in all patients (*n* = 6) as well as a co-localization of MAC with IgG3 could be detected (1/3 tested cases), there was no positive staining for IgG2 and IgG4. NDC did not stain positive for C5b-9 or any IgG subtype. C5b-9 staining = red, IgG subclasses = green, nuclear staining by DAPI = blue, double-positivity of C5b-9 and IgGs = yellow arrow, no double-positivity = white arrow, scale bar = 25 μm; motor endpoint regions with terminal nerve bundles (asterisk)

IgG subtyping via immunofluorescence on ICM tissue was performed in 5/7 LRP4-ab^+^ MG patients (insufficient biopsy material in two patients). All analysed LRP4-ab^+^ MG specimens showed co-localization of C5b-9 with IgG1 (Fig. 2a), with C5b-9 clearly located on NMJs, as visualized by additional co-staining of C5b-9 and α-Bungarotoxin (exemplary pictures of LRP4-ab^+^ MG and AChR-ab^+^ patient see Fig. 2b). One of the double-seropositive LRP4-ab/AChR-ab ^+^ MG patients (patient V) showed additional IgG3 staining on single endplates (see Table 2; Fig. 2a). There was no IgG2 or IgG4 staining at the NMJ of LRP4-ab^+^ MG patients. In AChR-ab⁺ MG, IgG1 and occasional IgG3 co-localized with C5b-9. No IgG subtype staining was observed in NDC samples (Fig. 3).

**Table 2:** Overview findings complement deposition and IgG-subtyping

Patient	Ab-status	C5b-9 (MAC)	IgG1	IgG2	IgG3	IgG4	Endplate destruction in EM
I	LRP4-ab+	+	+	-	-	-	One NMJ detected: Partial rarefication shortening and plumping of postsynaptic clefts
II	LRP4-ab+	+	n.d.	n.d.	n.d.	n.d.	Multiple NMJ detected: Clear rarefication, shortening and plumping of postsynaptic clefts
III	LRP4-ab+	+	n.d.	n.d.	n.d.	n.d.	No endplates detected
IV*	LRP4-/AchR-ab+	+	+	-	-	-	One NMJ detected: No overt alterations
V*	LRP4-/AchR-ab+	+	+	-	+	-	No endplates detected
VI	LRP4-ab+	+	+	-	-	-	n.d.
VII	LRP4-ab+	+	+	-	-	-	Multiple NMJs detected: Overt shortening and rarefication of postsynaptic clefts partially appearing as short stumps

ab=antibody, EM=electron microscopy, Ig=immunoglobulin, MAC=membrane attack complex, n.d. not done due to insufficient biopsy material, NMJ=neuromuscular junction

*patients with one-time low AChR-ab titer of 0.6 nmol/l, 0.7 nmol/l and 0.6 nmol/l, respectively

In 4/7 LRP4-ab⁺ patients, at least one NMJ could be evaluated by electron microscopy. Three of these four patients showed postsynaptic structural changes, including reduced and shortened postsynaptic clefts. One double-seropositive patient showed a single NMJ without observable abnormalities (for details see Table 2).

**Fig. 4:**
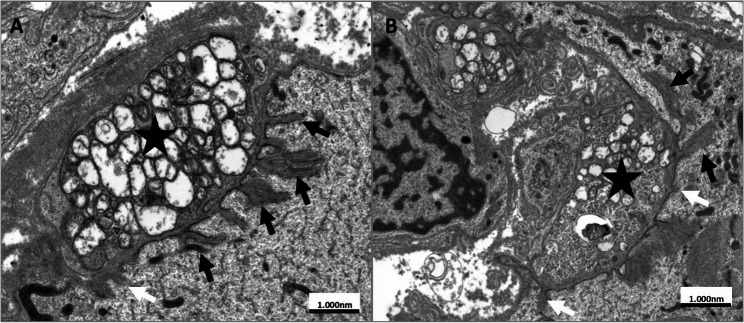
Ultrastructural analysis of endplate regions of patient VII. **a**, **b** (different NMJ of the same patient): Ultrastructural illustration of endplates with the terminal axon (bouton terminal) filled with mitochondria (asterisk). On the postsynaptic side, the clefts are scarce in number (black arrows), some of them are short and some plump (white arrow)

One LRP4-ab+-MG patient (patient VII) with C5b-9 deposition and marked postsynaptic ultrastructural changes on EM (see Table 2; Fig. 4) experienced a severe disease course, including myasthenic crisis (MGFA V), despite long-term immunosuppression with mycophenolate mofetil (azathioprine avoided due to allopurinol use). Plasma exchange led to temporary improvement, and additional treatment with intravenous immunoglobulin, rituximab (2 × 1000 mg), and efgartigimod (4 × 800 mg i.v.) did not result in sustained symptom stabilization. Following biopsy confirmation of C5b-9 deposition, treatment with the C5-complement inhibitor eculizumab (2 × 900 mg) was initiated and was associated with clinical improvement that allowed discharge from intensive care. Eculizumab therapy was continued thereafter.

## Discussion

Our study provides ‘*in situ’-*evidence that LRP4-ab^+^ MG patients show complement deposition at motor endplates and that the complement system is mainly activated via the classical IgG1-mediated pathway. Ultrastructural analysis suggests that LRP4-ab can mediate prominent endplate destruction.

All LRP4-ab^+^ MG patients assessed for IgG-subtyping showed co-localization of C5b-9 and IgG1, none stained positive for IgG2. IgG1 is a stronger complement activator than IgG2 and, together with IgG3, is among the strongest complement activator of all IgG-subclasses [2]. The ability of LRP4-ab to impair AChR-clustering at the postsynaptic membrane as their postulated main effector mechanism is supported by experimental data [22]. It has also been shown that serum samples of LRP4-ab^+^ MG patients can disrupt the Agrin-LRP4 interaction and inhibit AChR clustering [23]. However, this impairment of AChR clustering was not apparent in all LRP4-ab^+^MG patients, suggesting that different pathophysiological effector mechanisms such as complement activation might play a role in LRP4-ab^+^ MG, which is supported by our findings. Complement activation might also be specific for the muscular compartment, as one study showed no increase in serum levels of activated complement proteins in patients with LRP4-Ab^+^ MG compared with those detected in NDC, except for a marginal increase in C4a concentrations in LRP4-Ab^+^ MG [16]. Consistent with this compartment-specific perspective, complement transcript levels in our analyses did not differ significantly between groups. However, these data should be interpreted cautiously, as they derive from whole-muscle biopsies rather than isolated endplate regions. Accordingly, local complement activation at the NMJ may be detectable at the protein level without parallel differences in bulk muscle gene expression.

One double-seropositive patient showed additional IgG3 deposition at the NMJ. To date, there is no study indicating that LRP4-ab could be of the IgG3 subtype, whereas AChR-ab can. Our methods cannot identify the specific post-synaptic antigens targeted by the IgG subtypes, nor determine which antibody predominates in driving the clinical syndrome. Accordingly, in double-seropositive cases it cannot be determined whether complement activation is driven by LRP4 antibodies, AChR antibodies, or both. Therefore, this finding in a single patient should be interpreted with caution.

LRP4-ab remain a matter of debate, and their pathogenic relevance has been questioned in recent studies [14, 15]. Reported prevalence rates vary widely, and differences in assay type as well as in assay sensitivity and specificity contribute to these heterogeneous finding [5, 23–25]. In our cohort, AChR and MuSK-ab were detected by ELISA, a method in which low-titer reactivity may occasionally represent false-positive results [26]. Thus, low-titer AChR reactivity in three patients may not reflect true seropositivity. At the same time, the interpretation of LRP4-ab detected by F-CBA must consider the known variability of this method across studies. To ensure transparency of the detection method used here, Supplementary Fig. 1 provides representative negative and positive control patterns from the same L-CBA platform used for patient testing. Additional L-CBA and F-CBA testing of available archived sera confirmed AChR and MuSK seronegativity in most retested cases. One patient showed low positive MuSK reactivity only in the L-CBA, illustrating the assay dependent variability of antibody detection and the challenges of serological classification in rare MG subgroups.

Complement deposition at the NMJ has been demonstrated not only in AChR-ab⁺ MG, the classical complement-mediated subtype, but also in triple seronegative MG [27, 28]. These observations show that local complement activation is not confined to a single serological MG subgroup. Accordingly, although serological uncertainty cannot be fully excluded in individual cases, the combination of L-CBA-confirmed LRP4-ab positivity and the tissue-level findings presented here supports LRP4-related autoimmunity as a plausible explanation for the observed pathology.

Study limitations include the small sample size, which reflects the rarity of LRP4-ab⁺ MG as an orphan disease and limits the possibility of subgroup analyses. In addition, several patients were receiving immunosuppressive or immunomodulatory therapies at the time of biopsy. While these treatments could theoretically influence histopathological findings, data on their impact on complement deposition or ultrastructural NMJ alterations are currently very limited. Intercostal muscle biopsy provides reliable access to motor endplates but requires thoracic or orthopedic surgical procedures, reducing feasibility for broader application. Limb muscle biopsies are more accessible but often yield insufficient NMJs for meaningful analysis. These factors should be considered when interpreting the generalizability of our findings.

In conclusion, our data provide direct in situ evidence of complement activation at the NMJ in LRP4-ab⁺ MG and show that IgG1 is present at sites of C5b-9 deposition together with ultrastructural alterations. Given existing uncertainties regarding LRP4-ab pathogenicity and methodological limitations of serological testing, these findings contribute tissue-level context to an understudied MG subgroup. Further studies with larger cohorts are required to clarify the mechanistic role of LRP4-ab and to determine whether complement-targeted treatments may be beneficial in selected patients with biopsy-confirmed complement deposition.

## Supplementary Information

Below is the link to the electronic supplementary material.

Supplementary Material 1

## Data Availability

Anonymized data not published within this article will be made available by request from the corresponding author on reasonable request.
