# Supplementary material for: Complement deposition, IgG subtyping and endplate destruction in LRP4-ab-positive myasthenia gravis
Source: Acta Neuropathol Commun. 2026 Apr 21;14:102. doi: 10.1186/s40478-026-02284-0 (PMC13112662; doi:10.1186/s40478-026-02284-0)
Supplement: Supplementary file 1 — Supplementary Material 1 [file 40478_2026_2284_MOESM1_ESM.docx]

**Supplemental material**

**Title page**

Title: Complement deposition, IgG subtyping and endplate destruction in LRP4-ab-positive myasthenia gravis

Sarah Hoffmann (MD)^1^, Katharina Brokamp^1^, Andreas Meisel (MD)^1^, Paolo Doksani^1^, Lissy Helmig^1^, Markus Schuelke (MD)^2^, Jens-Carsten Rückert (MD)^3^, Matthias Pumberger (MD)^4^, Friederike Schömig (MD)^4^, Marlene Wolfsgruber^5^, Inga Koneczny^5^, Marie Mayrhofer^6^, Martin Blüthner^6^, Tobias Ruck (MD)^7,8,9^, Marc Pawlitzki (MD)^10^, Werner Stenzel* (MD)^11,^ Corinna Preusse (PhD)*^1,2,11^

*These authors contributed equally

^1^Charité – Universitätsmedizin Berlin, corporate member of Freie Universität Berlin and Humboldt Universität zu Berlin, Department of Neurology with Experimental Neurology, Charitéplatz 1, 10117 Berlin, Germany

^2^Charité – Universitätsmedizin Berlin, corporate member of Freie Universität Berlin and Humboldt-Universität zu Berlin, Department of Neuropaediatrics, Augustenburger Platz 1, 13353 Berlin, Germany

^3^Charité – Universitätsmedizin Berlin, corporate member of Freie Universität Berlin and Humboldt Universität zu Berlin, Department of Thoracic Surgery, Charitéplatz 1, 10117 Berlin, Germany

^4^Charité - Universitätsmedizin Berlin, Berlin, Germany Charité – Universitätsmedizin Berlin, corporate member of Freie Universität Berlin and Humboldt Universität zu Berlin, Center for Musculoskeletal Surgery, Charitéplatz 1, 10117 Berlin, Germany

^5^Division of Neuropathology and Neurochemistry, Department of Neurology, Medical University of Vienna, Vienna, Austria.

Department of Autoimmune Diagnostics, Medizinisches Versorgungszentrum (MVZ) ^6^Laboratory PD Dr. Volkmann & Colleagues SE & Co. eGbR, Karlsruhe, Germany

^7^Ruhr University Bochum, BG University Hospital Bergmannsheil, Department of Neurology, Bochum, Germany.

^8^BG University Hospital Bergmannsheil, Heimer Institute for Muscle Research, Bochum, Germany.

^9^Department of Neurology, Medical Faculty, Heinrich Heine University Duesseldorf; Duesseldorf, Germany.

^10^Department of Neurology, Medical Faculty and University Hospital Düsseldorf, Heinrich-Heine-University Düsseldorf, Düsseldorf, Germany

^11^Charité – Universitätsmedizin Berlin, corporate member of Freie Universität Berlin and Humboldt-Universität zu Berlin, Department of Neuropathology, Charitéplatz 1, 10117 Berlin, Germany

Corresponding author:

Sarah Hoffmann, M.D.

Department of Neurology, Charite – Universitätsmedizin Berlin, Germany

Charitéplatz 1

10117 Berlin

Phone: +49 30 450 639726

Fax: +49 30 450 539 913

Email: sarah.hoffmann@charite.de

**
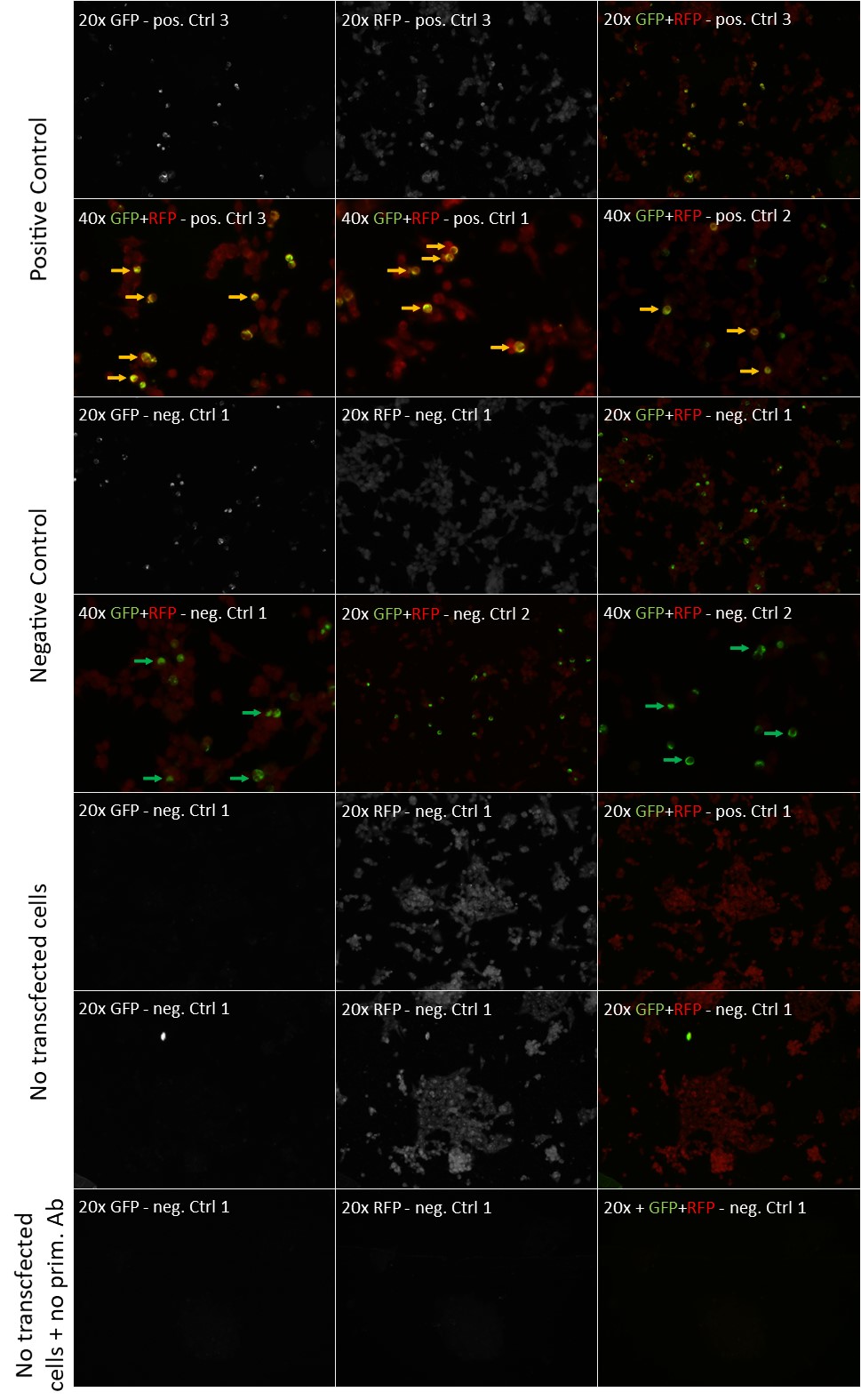
**

**Supplementary Fig 1 Representative fluorescence patterns of the fixed cell–based assay (L-CBA) used for LRP4-antibody detection**

HEK293 cells transiently expressing GFP-tagged human LRP4 appear in green. Patient sera are incubated with these cells, and bound human IgG is visualized with an RFP-conjugated secondary antibody (red). Co-localization of GFP and RFP signals produces a yellow fluorescence signal in the merged images, indicating antibody binding to LRP4-expressing cells. To ensure accurate identification of anti-LRP4 antibody–positive sera, two criteria were applied. First, LRP4 is a transmembrane protein and therefore localizes to the cell membrane, producing a characteristic membranous staining pattern in cells incubated with LRP4 antibody-positive sera. Second, cells were transfected with a plasmid encoding intact GFP-coupled LRP4. GFP fluorescence serves as a marker of successfully transfected cells and indicates the localization of expressed LRP4. Sera were considered positive only when overlapping red fluorescence and green fluorescence (GFP) colocalized in the expected membranous pattern, allowing clear identification of double-positive cells. Negative controls show isolated GFP fluorescence without RFP signal. Staining performed in the absence of primary antibody showed no signal. These images illustrate the reference patterns used for interpreting LRP4-antibody reactivity in the assay.

Pictures with permission and from: Bluethner, Martin, Mayrhofer, Marie and Wohlrab, Melanie; Department of Autoimmune Diagnostics, Medizinisches Versorgungszentrum (MVZ) Laboratory PD Dr. Volkmann & Colleagues SE & Co. eGbR, Karlsruhe, Germany


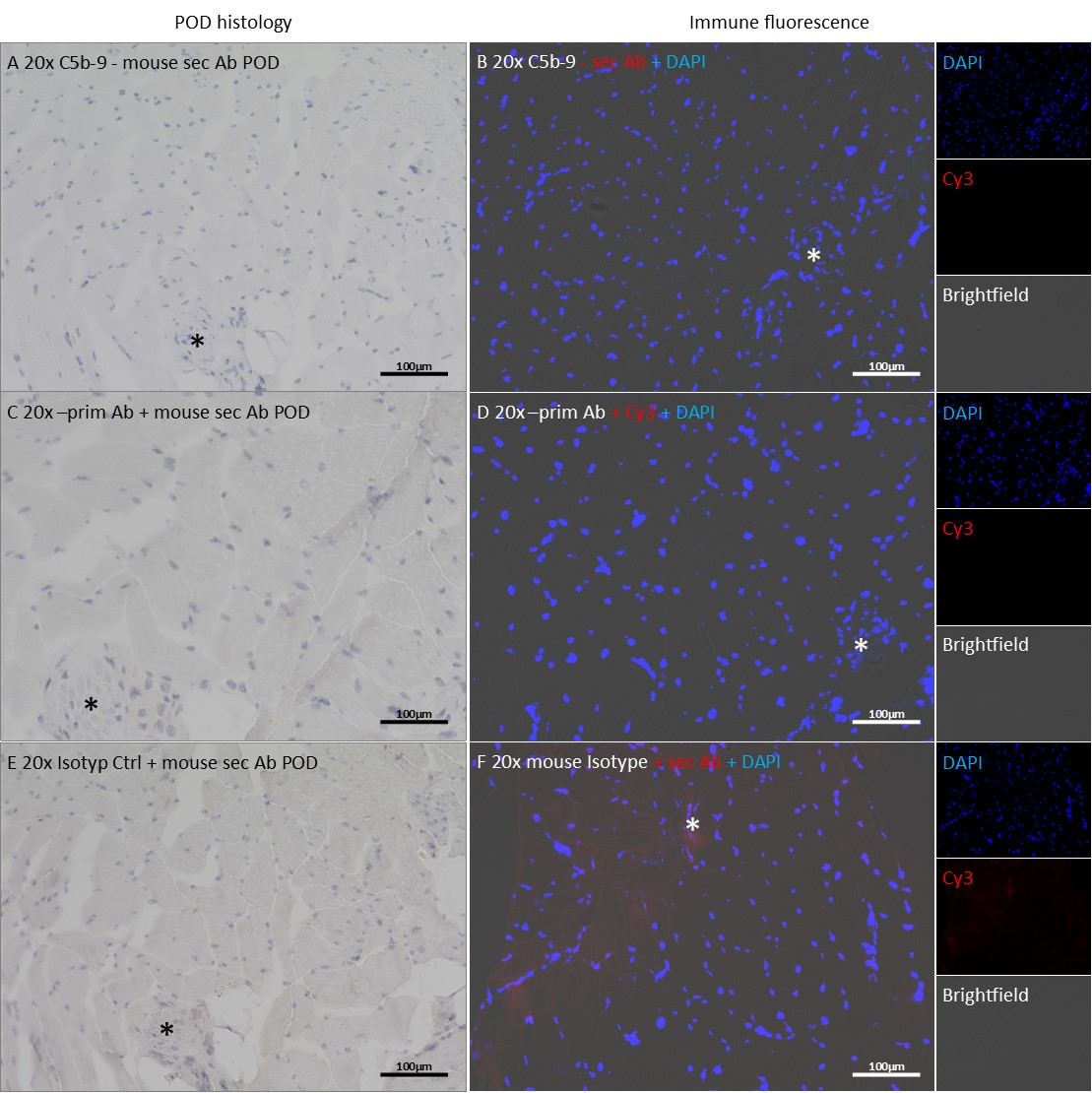


**Supplementary Fig 2 Representative images of negative control staining in human skeletal muscle tissue**

Representative images of human skeletal muscle sections processed under negative control conditions for histology (A, C, E) and immunofluorescence (B, D, F). Histological staining controls: (A) primary antibody only (no secondary antibody), (B) primary antibody omitted with secondary antibody applied, and (C) isotype control antibody with secondary antibody, same concentration as C5b-9, 1µg/ml. (D–F) Immunofluorescence controls performed under the same conditions: (D) primary antibody only (no secondary antibody), (E) primary antibody omitted with secondary antibody (Cy3) applied, and (F) isotype control antibody with secondary antibody Cy3. All conditions showed absence of specific chromogenic signal or fluorescence, confirming lack of nonspecific staining in human muscle tissue. Scale bar = 100µm. Asterisk = nerve fascicle.
